# Supplementary material for: Learning curve and surgical outcome of robotic assisted colorectal surgery with ERAS program
Source: Sci Rep. 2022 Nov 29;12:20566. doi: 10.1038/s41598-022-24665-w (PMC9709162; doi:10.1038/s41598-022-24665-w)
Supplement: Supplementary file 1 — Supplementary Information 1. [file 41598_2022_24665_MOESM1_ESM.zip › LSC Raw data-Ñ[▒K/139/N_ñw╝╨░O▒Kñσ.pdf]

W83 / W83

臺中榮民總醫院
手術室護理記錄

[redacted]

基本資料

- 室別： 開刀房-18室，手術科別： CRS，手術類別： 常規，日期： 2021/10/01
- 進入時間： 0740 ； 麻醉開始時間： 0745 ； Time Out時間： 0900 ； 劃刀時間： 0901 ； 手術結束時間： 1230 送出時間： 1250 ；
- 麻醉方式： GE
  診斷：Malignant tumor of cecum
  手術名稱：Laparoscopic right colectomy and anastomosis
  手術局部用藥：NIL.
  術前預防性抗生素：
  - 抗生素名稱一： Veterin 1GM IVD由麻醉護理師給予
  - 時間： 0840
  - 上次簽章: 張秀敏

  ，追加預防性抗生素：
- 手術醫師： 蔣鋒帆，邱騰逸，陳映菁，陳凱祥
- 麻醉醫師： VS 沈靜慧
-
  - 刷手護士A ： 黃鈺雯，時間： 0740，接替刷手一： 李苓瑜，時間： 1200
  - 流動護士A ： 張秀敏，時間： 0740，接替流動一： 林裕瑋，時間： 1200，接替流動二： 黃鈺雯，時間： 1230

皮膚狀況

A-V Shunt： 無
術前： 完整
　記錄者： 上次簽章: 張秀敏
術後： 完整
　記錄者： 上次簽章: 黃鈺雯

手術準備

手術臥式 單一手術臥式

-
  - 手術臥式： 仰臥
  - 使用附件： 溫毯 38 ℃，頭圈，其它： 矽膠墊
  - 皮膚準備： 10%Aq Betadine，Hibiscrub，2% Chlorhexidine
  - 電燒： 使用電燒: 電極片位置： 右大腿，記錄者： 上次簽章: 張秀敏

止血帶T： 無

植入物

無

標本送檢

- 細菌培養 無
- 細胞學 無
- 冰凍切片 無
- 病理標本
  組織名稱一：A COLON
  ,
  組織名稱二：uterus Peritoneum
- 截肢 無

標本交付 標本： 1 袋，標本： 0 盒，標本： 1 瓶，細胞學： 0 管，Culture Tube： 0 管，被交付醫師： Fellow 陳映菁，

- 記錄者： 上次簽章: 張秀敏
- 時間： 1208

傷口術後數料

傷口數料： 傷口數料： 無

- 請於圖上標示部位：

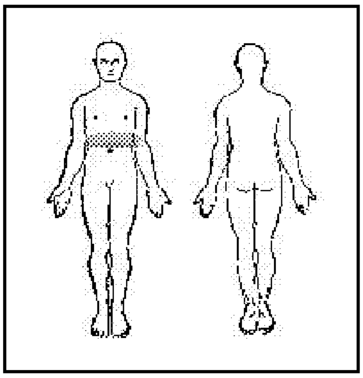

- 傷口數料： 紗布

導管

導管	天數	導管資訊	插入日期	插入時間	更換日期	拔除日期	拔除時間	拔除原因	預計拔除/更換	預計拔除/更換日期
Foley(1)	1	號碼:18 Fr.; 部位:膀胱;	2021/10/01	0820						
Sump NG Tube(1)	1	號碼:16 Fr.; 部位:胃;	2021/10/01	0855						
Epidural PCA(1)	1	部位:硬脊膜;	2021/10/01	0800						

用物計數

- 手術中出血量： minimal c.c.
- 手術中尿量： 100 c.c.
-
    - 其它： 無

用物計數： 單一切口

- 1.器械： 正確，2.縫針： 正確，3.刀片： 正確，4.紗布： 正確，5.腹部墊： 未使用，6.紗球： 未使用，7.PEANUTS ： 未使用，8.紗條： 未使用，9.絲帶： 未使用，10.針頭： 未使用，11.Vessel loop： 未使用，12.Boots： 未使用，13.Jelco： 未使用，14.棉條： 未使用，
    - 第一次：
        - 刷手護士 ： 黃郁雯
        - 流動護士： 上次簽章: 張秀敏
        - 時間： 1150

  ,
    - 第二次：
        - 刷手護士 ： 黃郁雯
        - 流動護士： 上次簽章: 張秀敏
        - 時間： 1155

  ,
    - 第三次：
        - 刷手護士 ： 李苓瑜
        - 流動護士： 上次簽章: 張秀敏
        - 時間： 1205

備註

備註： 第6室腹腔鏡NO. 1

- 記錄者： 上次簽章: 張秀敏
- 時間： 0800

填寫護理人員 黃鈺雯

正式摘要

W83 / W83

doctorRank:B
